# Concordance of Expert and Parental Opinion about Hypospadias Surgical Outcome Is Severity Dependent

**DOI:** 10.3389/fped.2016.00002

**Published:** 2016-01-25

**Authors:** Marcos Raymond Pérez-Brayfield, Juan Carlos Jorge, Luis A. Avilés, Joanne Díaz, Vanessa Ortiz, Wilfredo Morales-Cosme

**Affiliations:** ^1^Division of Urology, School of Medicine, University of Puerto Rico, San Juan, PR, USA; ^2^Department of Anatomy and Neurobiology, School of Medicine, University of Puerto Rico, San Juan, PR, USA; ^3^Department of Social Sciences, School of Public Health, University of Puerto Rico, San Juan, PR, USA; ^4^MD Program, School of Medicine, University of Puerto Rico, San Juan, PR, USA; ^5^MPH Program, School of Public Health, University of Puerto Rico, San Juan, PR, USA

**Keywords:** hypospadias, hypospadias severity, genital surgery, surgical outcomes, surgery satisfaction, kappa coefficient

## Abstract

**Introduction:**

Hypospadias is a male congenital condition where the opening of the urethral meatus is not located in the typical anatomical position. It has been a challenge for empirical studies to ascertain the level of concordance of opinion among parents and urologists with regard to surgical outcomes according to hypospadias severity.

**Materials and methods:**

Parents of children who had undergone hypospadias repair were recruited for this study (*n* = 104). A set of questionnaires that included some items with Likert scale were created to evaluate postsurgical satisfaction by parents and urologists. SAHLSA-50, a validated instrument for adult Spanish-speaking adults, was used to assess health literacy. Cohen’s kappa (κ) coefficient was used to assess interobserver agreement and Chi Square “Goodness of Fit” Test was used to determine probability of satisfaction.

**Findings:**

Concordance on the level of satisfaction with surgical outcomes for Type cases I was slight (κ = 0.20; CI 95% 0–0.60), for Type II cases was moderate (κ = 0.54; CI 95% 0.13–0.94), and for Type III cases was substantial (κ = 0.62; CI 95% 0–0.56). However, the probability of satisfaction did not change according to severity (Chi Square “Goodness of Fit” Test; parents, *p* = 0.84; pediatric urologists, *p* = 0.92). These results cannot be explained by parental health literacy according to SAHLSA-50 test scores.

**Conclusion:**

The level of concordance of opinion among parents and urologists with regard to their level of satisfaction with surgical outcomes is related to hypospadias severity, whereby the greatest level of concordance of opinion was achieved among most severe cases. This study underscores the need for longer follow-up to properly assess satisfaction with hypospadias repair, especially for the less severe forms of the condition.

## Introduction

Hypospadias refers to the atypical positioning of the urethral meatus in males. However, cases can be quite diverse, including atypical shape of the urethral opening, of the glans and penile skin, various degrees of penile curvature, penoscrotal transposition among other variations in genital appearance. Severity of the condition refers to the position of the opening of the urethral meatus that can range from a glanular or subcoronal position (Type I), midshaft position (Type II), to a proximal, penoscrotal, scrotal, or perineal position (Type III) (1).

There is great need for standardized algorithms for outcome assessment after hypospadias repair (2–4). In fact, little is known about the level of concordance of opinion between the pediatric urologist and parents in outcome assessment. Outcome assessment can be classified under four broad domains: postoperative complications, cosmetic appearance of penis, functional outcome (micturition and sexuality), and quality of life and psychosexual life (4). However, it is difficult to assess functional outcome in non-toilet-trained infants. Similarly, assessment of psychosexual life is not applicable to pediatric patients. For these patients, parents are the ultimate judges of outcome assessment after hypospadias repair of their child. Nevertheless, there is a paucity of pediatric urology studies that address the level of satisfaction with surgical repair of hypospadias among parents and physicians within a sample of patients. In this study, we aimed to contribute to the field by assessing the level of concordance on surgery satisfaction between parents and physicians after hypospadias repair according to the severity of the condition.

We hypothesized that parents of children born with severe hypospadias would be less satisfied with surgical outcomes than parents of children born with less severe forms of the condition. In order to address this hypothesis, parents of children born with hypospadias were recruited from three pediatric urology clinical sites. Likert scales were used to assess parental and urologist’s level of satisfaction.

## Materials and Methods

### Sample and Data Collection Instruments

Parents of children with hypospadias were recruited for this study under the direct supervision of one of the coauthors (Marcos Raymond Pérez-Brayfield; *n* = 104 cases). Outcome assessment in this study was assessed for the period from October 2012 to June 2015. All patients underwent either a pyramid or Snodgrass repair and had at least 6 months of postoperative follow-up at the time of evaluation. Hypospadias severity of the child was confirmed at clinical sites. Questionnaires were used to inquire about the health status of child, mother, and father. Health literacy was assessed with the SAHLSA-50 test (5), which is a validated instrument for adult Spanish-speaking adults. Questionnaires were used to evaluate postsurgical satisfaction by parents and urologists.

Evaluation of surgical outcomes was assessed with a direct question: “are you satisfied with the results of the surgery?” (in Spanish language: *“¿Estás satisfecha/o con los resultados de la cirugía?*”) with answers provided in a Likert scale: “very satisfied, satisfied, unsatisfied, very unsatisfied” (in Spanish language: “*Muy satisfecho, Satisfecho, Insatisfecho, Muy insatisfecho*”). In order to minimize bias in these four levels of opinion between parental and expert’s opinion (Marcos Raymond Pérez-Brayfield), data were pooled according to being satisfied (very satisfied and satisfied) versus not being satisfied (unsatisfied and very unsatisfied) with surgical outcomes. Inclusion criteria did not require both parents to participate in this study. Nevertheless, parental evaluation of surgical outcomes is interpreted as a consensus of opinion between both parents.

### Statistics

Cohen’s kappa (κ) coefficient (6) was used to assess interobserver agreement and Chi Square “Goodness of Fit” Test was used to determine probability of satisfaction.

### Ethics Committee

This study received approval from the Institutional Review Board (protocol number A9000112). Personal identifiers from study participants were not collected.

## Results

**Figure 1:**
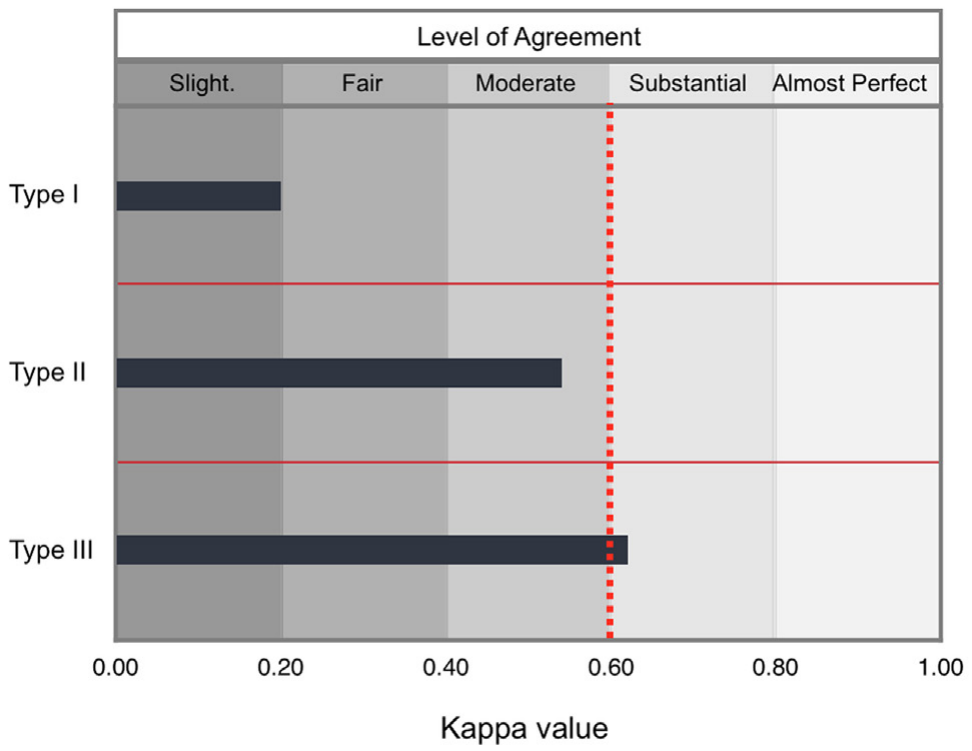
**Level of concordance among parents and urologists with regard to the level of satisfaction with surgical outcomes according to severity**. Concordance on surgery satisfaction among parents and the pediatric urologist after hypospadias repair according to kappa scores was “slight” for Type I cases (*n* = 52), “moderate” for Type II cases (*n* = 21), and “substantial” for Type III cases (*n* = 31). Ranges for kappa values for each level are shown. The dotted red line demarcates the target value that is minimum for substantial concordance.

There is a relationship between concordance on the level of satisfaction with hypospadias repair among parents and urologists versus the severity of the condition. Specifically, concordance for Type I cases was slight (κ = 0.20; CI 95% 0–0.60), for Type II cases was moderate (κ = 0.54; CI 95% 0.13–0.94), for Type III cases was substantial (κ = 0.62; CI 95% 0–0.56). Figure 1 shows the relationship between hypospadias severity and concordance on the level of satisfaction with surgical outcomes; from slight to substantial concordance for Type I through Type III cases. Following the Chi Square “Goodness of Fit” Test, we found that the probability of satisfaction did not change by severity (data not shown; parents, *p* = 0.84; pediatric urologists, *p* = 0.92). Postsurgical complications were infrequent: meatal stenosis for Type I (*n* = 1) and fistula for Type II (*n* = 1) and Type III (*n* = 2). All patients with Clavien III-b complications had revision surgery with favorable outcomes.

**Figure 2:**
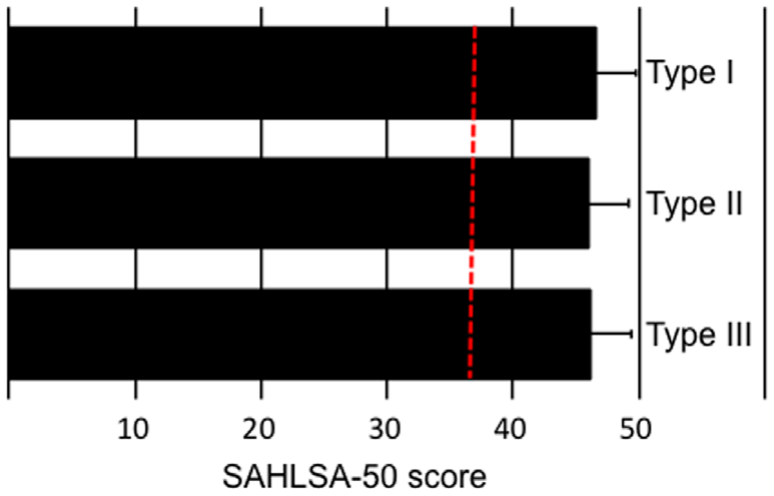
**Parental health literacy according to hypospadias severity of their child**. According to the validated health literacy test for Spanish-speaking adults, SAHLSA-50, there is no difference in health literacy among parents according to severity of their child. The dotted red line demarcates the minimum score that is required for an adequate health literacy level (score of 37).

According to SAHLSA-50 test scores, these differences in surgery satisfaction after hypospadias repair cannot be explained by differences in parental health literacy among Spanish-speaking parents (Figure 2).

## Discussion

We found a linear relationship between concordance of opinion among parents and urologists with regard to the level of satisfaction with surgical outcomes and hypospadias severity, where the level of concordance was greatest for most severe cases. Current standards of care for hypospadias are guided by a plethora of surgical techniques, most of which have improved over the years (7). Although the level of experience of the surgeon with such techniques is important (8–10), parents are the ultimate judges of surgical outcomes. It is worrisome that parents of children born with less severe forms of the condition may not have realistic expectations on what surgical interventions can accomplish. It is important to underscore the need for studies that specifically address the plausible links between parental education and their assessment of surgical outcomes after hypospadias repair. In this study, all parents were at least high school graduates. By using a simple standardized test that measures health literacy among Spanish-speaking adults (SAHLSA-50), we were unable to detect differences in health literacy scores according to the level of satisfaction. Similarly, there is a need to establish whether socioeconomic status can impinge upon parental perception of surgical outcomes after repair. So far, we have been unable to detect social class differences among households across severity cases even though we include variables other than traditional measures of socioeconomic status (data not shown).

It remains as a challenge for future studies to ascertain which aspects of the surgical repair are most important for parents according to severity. For instance, the weight that cosmetic versus functional outcomes has in determining the level of surgical satisfaction ought to be determined. Following our results, it is plausible that parental criteria depend on the severity of the condition, whereby functionality of the urogenital system in severe cases outweighs cosmetic considerations (11, 12). By using the Chi Square “Goodness of Fit” Test, we found that the probability of satisfaction did not vary as a function of severity. This complex clinical scenario indicates that, although parents may use different sets of criteria, the severity of the condition by itself is not a predictor of expert or parental opinion about surgical outcome.

There are a number of validated instruments that measure satisfaction with surgical outcomes after hypospadias repair. A recent study with a small sample of pediatric patients shows that the Pediatric Penile Perception Scale (PPPS) scores correlate with hypospadias severity but not the Hypospadias Objective Scoring Evaluation (HOSE) scores (13). A recent long-term follow-up study with a sample of 167 hypospadiac patients with a mean age of 34 shows that patients with the most severe forms of the condition were less satisfied with cosmetic and functional outcomes than those with milder forms as measured with PPPS translated to Swedish (14). Another study found satisfactory long-term outcomes as measured with HOSE and PPPS (15).

In the case of pediatric patients, it has been shown that postoperative complications, parental desire to avoid circumcision and initial decisional conflict level are predictors of parental decisional regret after hypospadias repair of their children (16). For the case of distal hypospadias repair, a more recent study found that factors unrelated to the surgical procedure in itself – parental educational level, not being first born, family history of hypospadias, initial desire to avoid surgery, younger age at follow-up, presence of lower urinary tract symptoms, and lower PPPS scores – are associated with parental decisional regret (17). Therefore, it is of interest for future work to assess the reasons for parental dissatisfaction and to address the relationships between decisional regret and parental satisfaction with hypospadias repair according to severity.

There is a growing interest in shared decision-making (SDM) as a research area, particularly as it applies to pediatric care (18). A recent meta-analysis study concluded that although SDM may improve medical knowledge and may decrease decisional conflict, it does not seem to improve satisfaction with the medical procedure (19). Jones et al. (20) conducted a prospective study, whereby adolescent patients were inquired about their satisfaction with hypospadias repair while their parents were asked to evaluate the quality of life of their children and the health care system in itself. Nevertheless, the missing link that still needs to be addressed is to study the relationships between parental satisfaction with hypospadias repair and patient satisfaction with surgical outcomes later in life. In this context, a better understanding of this link may improve the levels of parental and patient satisfaction with surgical outcomes across severities.

## Conclusion

We have shown that hypospadias severity is related to the level of concordance among parents and the pediatric urologist with regard to the level of satisfaction with surgical outcomes; the greatest level of concordance of opinion was achieved among most severe cases. This study underscores the need for longer follow-up to properly assess satisfaction with hypospadias repair. Therefore, clinical algorithms that involve surgical management of hypospadias must take severity of the condition into consideration when accompanying parents through difficult treatment decisions because agreeing to disagree is not an option.

## Author Contributions

MP-B and JJ: conceived, designed, and supervised the execution of the study. JD, VO, WM-C: acquired, analyzed, and interpreted data. LA: provided recommendations on study design and conducted data analyses. MP-B and JJ: drafted the first version of the manuscript, and all authors approved the final version that was submitted for publication. All authors are accountable for all aspects of this work.

## Conflict of Interest Statement

The authors declare that the research was conducted in the absence of any commercial or financial relationships that could be construed as a potential conflict of interest.
